# Impact of a Major Earthquake on Glycemic Control in Adults with Type 2 Diabetes: A Retrospective Cohort Study from Türkiye

**DOI:** 10.3390/diagnostics15182361

**Published:** 2025-09-17

**Authors:** Sedat Ozdemir, Sadettin Ozturk

**Affiliations:** 1 Department of Internal Medicine, School of Medicine, Gaziantep Islam Science and Technology University, 27010 Gaziantep, Turkey; 2 Department of Endocrinology and Metabolism, Gaziantep Dr Ersin Arslan Training and Research Hospital, 27090 Gaziantep, Turkey; sadettinozturk27@hotmail.com

**Keywords:** type 2 diabetes mellitus, earthquakes, glycemic control, hemoglobin A1c, natural disasters

## Abstract

**Objectives:** This study aimed to evaluate the impact of the 6 February 2023 Kahramanmaraş-centered earthquakes on glycemic control in adults with type 2 diabetes mellitus (T2DM) by analyzing pre–post changes in HbA1c and fasting glucose. In addition, it sought to identify key clinical and biochemical predictors of glycemic worsening in the post-disaster period using routinely available laboratory data. **Materials and Methods:** This retrospective pre–post observational cohort study included 550 adult patients with established T2DM who received care at two centers in Gaziantep, Türkiye. Laboratory data—including HbA1c, fasting glucose, triglycerides, HDL, and albumin—were compared between two periods: three months before and three to five months after the earthquake. Paired samples *t*-tests, Spearman’s correlation, multiple linear regression, and ROC analysis were used to evaluate changes in glycemic control and its predictors. **Results:** The mean age of the participants was 56.2 ± 11.0 years; 43.3% were male and 56.7% female. Post-earthquake, HbA1c (*p* = 0.012) and fasting glucose (*p* < 0.001) increased significantly, indicating deterioration in metabolic control. White blood cell (*p* = 0.003) and platelet counts (*p* < 0.001) rose, while HDL (*p* < 0.001), ALT (*p* = 0.002), and triglycerides (*p* = 0.010) decreased. ΔHbA1c correlated positively with ΔGlucose (r = 0.362, *p* < 0.001), ΔTriglyceride (r = 0.323, *p* < 0.001), LDL (r = 0.173, *p* < 0.001), and total cholesterol (r = 0.107, *p* = 0.032), and negatively with ΔAlbumin (r = −0.332, *p* = 0.029), ΔHDL (r = −0.175, *p* < 0.001), and WBC (r = −0.112, *p* = 0.009). In the fully adjusted multivariable model (age, sex, BMI, diabetes duration, insulin use), independent predictors of ΔHbA1c included ΔGlucose (β = 0.007, *p* < 0.001), ΔTriglyceride (β = 0.004, *p* = 0.001), ΔHDL (β = −0.010, *p* = 0.011), ΔAlbumin (β = −0.016, *p* = 0.007), and ΔWBC (β = −0.009, *p* = 0.022). Clinical predictors were BMI (β = 0.006, *p* = 0.045), diabetes duration >10 years (β = 0.094, *p* = 0.009), and insulin use (β = 0.121, *p* = 0.003) (Adjusted R^2^ = 0.319). ROC analysis confirmed ΔGlucose as the strongest predictor of worsening glycemic control (AUC = 0.81; sensitivity 82.1%, specificity 73.4%). Additional predictors included insulin use (AUC = 0.66, *p* < 0.001), ΔTriglyceride (AUC = 0.65, *p* < 0.001), BMI (AUC = 0.63, *p* = 0.002), diabetes duration >10 years (AUC = 0.62, *p* = 0.004), and ΔHDL (AUC = 0.61, *p* = 0.016), each providing more modest predictive value. **Conclusions:** Glycemic control became significantly worse in adults with T2DM after the February 2023 earthquake, as reflected by increases in HbA1c and fasting glucose. Both biochemical parameters (Glucose, Triglyceride, HDL, Albumin, WBC) and clinical characteristics (BMI, diabetes duration >10 years, insulin use) were independently associated with glycemic deterioration. Among these, glucose remained the strongest predictor (AUC = 0.81), while BMI, insulin therapy, and longer diabetes duration provided additional predictive value. These findings suggest that routinely available clinical and laboratory data can be used to identify patients at highest risk of metabolic decompensation in disaster settings.

## 1. Introduction

Natural disasters such as earthquakes not only cause devastating physical destruction but also exert profound effects on the psychological and physiological well-being of affected populations. Among these, individuals with chronic illnesses are particularly vulnerable due to the disruption of healthcare services, limited access to medications, and elevated stress levels [1,2,3]. The Kahramanmaraş-centered twin earthquakes that struck southeastern Türkiye on 6 February 2023 affected over 13 million people across 11 provinces, resulting in unprecedented destruction and displacement, and placed a significant burden on the national healthcare infrastructure [4,5,6,7].

Type 2 diabetes mellitus (T2DM) is a chronic metabolic disorder characterized by insulin resistance and progressive beta-cell dysfunction, necessitating continuous monitoring and treatment [8]. Glycemic control in individuals with T2DM is influenced by a delicate balance of factors, including medication adherence, dietary regulation, physical activity, and psychosocial stability. In disaster settings, this balance is often disrupted [9]. Psychological stress can activate the hypothalamic–pituitary–adrenal (HPA) axis, increasing levels of cortisol and catecholamines, which in turn can impair insulin sensitivity and promote hepatic gluconeogenesis, exacerbating hyperglycemia [10,11]. Moreover, displacement, irregular meals, and loss of access to medications and routine follow-ups further deteriorate glycemic control [12].

Several studies following catastrophic events—such as the 2011 Great East Japan Earthquake, the 2018 Northern Osaka Earthquake, and Hurricane Katrina in 2005—have consistently reported worsened glycemic indices in individuals with diabetes during the post-disaster period [13,14,15,16]. These studies emphasize not only the metabolic consequences but also highlight the inequalities in healthcare access that become more pronounced during crises. However, while such evidence exists, most reports focus on type 1 diabetes or pediatric populations, and few offer detailed biochemical analysis of T2DM patients, particularly in middle-income countries such as Türkiye [12,17,18,19].

Despite the high prevalence of T2DM in Türkiye and the significant impact of the February 2023 earthquakes, there remains a paucity of studies examining how such large-scale disasters influence metabolic outcomes in adults with type 2 diabetes. Identifying patterns of glycemic deterioration and their potential associations with other metabolic parameters is crucial for guiding disaster preparedness strategies that are inclusive of chronic disease management.

It should also be recognized that poor diabetes management in general can independently lead to worsening glycemic control. However, in disaster settings, this baseline vulnerability may be further aggravated by disrupted healthcare access, medication discontinuity, and heightened psychosocial stress.

This study aimed to evaluate the impact of the 6 February 2023 earthquakes on glycemic control in adults with T2DM by analyzing pre- and post-disaster biochemical data, particularly focusing on hemoglobin A1c (HbA1c) and fasting glucose levels. In addition, we sought to identify clinical and biochemical predictors of worsening glycemic control in the post-disaster period. By identifying the metabolic consequences of disaster exposure, this research seeks to contribute to the growing body of evidence supporting the integration of chronic disease care into national disaster preparedness and response frameworks. We focused on routinely available laboratory markers such as WBC, albumin, triglycerides, and HDL, as these parameters are inexpensive, universally measured in clinical practice, and feasible for rapid use in disaster settings where advanced tests may not be accessible.

## 2. Materials and Methods

### 2.1. Study Design

This retrospective, multicenter, pre–post observational cohort study was conducted to investigate the effects of a major earthquake on glycemic control in patients diagnosed with type 2 diabetes mellitus (T2DM). The study included patients treated at two healthcare facilities under the Gaziantep Provincial Health Directorate: Ersin Arslan Training and Research Hospital and Islahiye State Hospital. Both centers are located in the southeastern region of Türkiye, one of the most affected areas during the Kahramanmaraş-centered earthquakes on 6 February 2023. The study period spanned from 1 April to 30 July 2024.

Ethical approval for this study was obtained from the Non-Interventional Clinical Research Ethics Committee of Gaziantep University Medical Faculty (Approval No: 2023/271; Date: 1 November 2023). The study was carried out in accordance with the Declaration of Helsinki and national regulations. All data were extracted in anonymized form from hospital records, and no identifiable personal information was collected or analyzed. As the study utilized anonymized data without direct patient contact, the requirement for individual informed consent was waived by the Ethics Committee.

### 2.2. Study Population and Inclusion Criteria

A total of 731 adult patients (≥18 years) with a confirmed diagnosis of T2DM were identified in the hospital records during the study period. After applying the predefined eligibility criteria, 550 patients were included in the final analysis.

The inclusion criteria were the following:

⮚ Documented T2DM diagnosis in the hospital information system prior to 6 February 2023;
⮚ Availability of at least one recorded HbA1c measurement and accompanying biochemical tests during both the three months before and the three to five months after the earthquake;
⮚ Residency in the disaster-affected region.

The exclusion criteria were the following:

⮚ Type 1 diabetes mellitus;
⮚ Gestational diabetes;
⮚ Secondary diabetes (e.g., pancreatogenic or steroid-induced diabetes);
⮚ Acute metabolic complications (e.g., diabetic ketoacidosis [DKA], hyperosmolar hyperglycemic state [HHS]);
⮚ Missing or incomplete laboratory data;
⮚ Residency outside the disaster zone;
⮚ Pregnancy, severe acute infection, or active malignancy at the time of testing.

After exclusions (*n* = 181), the final sample comprised 550 eligible patients, who were included for statistical analysis.

### 2.3. Data Collection

Patient data were retrospectively extracted from the Health Information Systems (HIS) of both hospitals. No additional diagnostic tests or follow-up visits were initiated for research purposes. The collected variables included the following:

✓ Demographics: age, sex, nationality, and residential location (central Gaziantep or Islahiye).
✓ Laboratory parameters (pre- and post-earthquake): HbA1c (%), fasting plasma glucose (mg/dL), total cholesterol, low-density lipoprotein (LDL), high-density lipoprotein (HDL), triglycerides, albumin, creatinine, blood urea nitrogen (BUN), alanine aminotransferase (ALT), aspartate transaminase (AST), white blood cell count (WBC), platelet count (PLT), hemoglobin, erythrocyte sedimentation rate (ESR), C-reactive protein (CRP), vitamin D, vitamin B12, and thyroid-stimulating hormone (TSH).

Both centers belong to the Gaziantep Provincial Health Directorate and use harmonized standard operating procedures for specimen collection, processing, and reporting. Pre-analytical conditions were uniform across sites: venous samples were obtained after 8–12 h of fasting; HbA1c was measured from EDTA whole blood; chemistry panels were performed on serum separated within routine turnaround times, and complete blood counts were analyzed from EDTA whole blood. Analytical methods were harmonized as follows: HbA1c was reported in NGSP (%) units with traceability to IFCC, measured using high-performance liquid chromatography (HPLC, Tosoh G8 Analyzer, Tosoh Bioscience, Tokyo, Japan); fasting plasma glucose was measured using a validated enzymatic method; lipid parameters (total cholesterol, LDL, HDL, triglycerides) were determined using enzymatic colorimetric assays; albumin was measured using a bromocresol-based dye-binding method; and hematological indices (WBC, PLT, hemoglobin) were obtained using automated hematology analyzers. Calibration and internal quality control were performed according to manufacturer recommendations and institutional policy, and both laboratories participated in external proficiency testing programs mandated by the health authority. Units and reference intervals were identical across sites via the shared laboratory information system. Because our statistical approach used paired, within-patient pre- versus post-earthquake measurements, the risk of inter-site analytical bias affecting the main outcomes was minimized.

**Figure 1:**
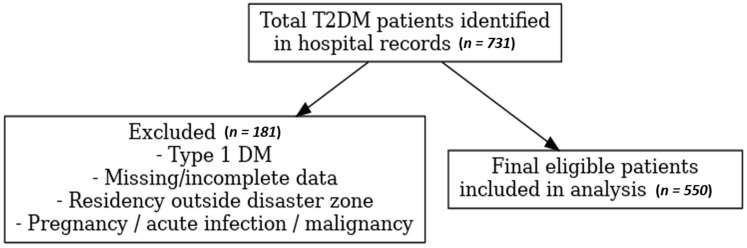
Flowchart of the study.

The ‘pre-earthquake’ period was defined as 1 November 2022 to 5 February 2023, and the ‘post-earthquake’ period as 15 March to 15 July 2023. The three to five-month post-disaster timeframe was chosen to allow for stabilization of acute disruptions immediately following the earthquake while still reflecting medium-term glycemic changes, as HbA1c represents an average of the preceding 8–12 weeks. Accordingly, the worsening glycemic control we report reflects changes that became evident within three to five months after the earthquake. Patients with laboratory results from both periods were included to allow for paired comparison (Figure 1).

### 2.4. Statistical Analysis

All statistical analyses were performed using IBM SPSS Statistics version 27.0 (IBM Corp., Armonk, NY, USA) and Python 3.10 for advanced modeling and visualization. Descriptive statistics were calculated for all demographic and clinical variables. Continuous variables were expressed as mean ± standard deviation (SD) or median (interquartile range) based on distributional characteristics, while categorical variables were presented as frequencies and percentages. The Kolmogorov–Smirnov test was used to assess the normality of continuous variables. To evaluate changes in laboratory parameters before and after the earthquake, paired samples *t*-tests were used for normally distributed variables. For confirmation, Wilcoxon signed-rank tests were additionally applied for all parameters, and the results were consistent across methods. A *p*-value of less than 0.05 was considered statistically significant. To explore associations between changes in glycemic control and alterations in other laboratory parameters, Spearman’s rho correlation analysis was performed using the change in HbA1c (ΔHbA1c) as the dependent variable. This correlation analysis served as the univariate screening step. Variables showing significant associations in univariate testing were subsequently included in a multiple linear regression analysis to identify independent predictors of ΔHbA1c. The strength of the model was assessed using R^2^ and adjusted R^2^ values, with beta coefficients and *p*-values reported for each variable. In addition, a Receiver Operating Characteristic (ROC) analysis was conducted to assess the predictive performance of key laboratory markers (ΔGlucose, ΔTriglyceride, ΔHDL) in identifying patients with significant glycemic worsening (defined as ΔHbA1c > 0.5%). In line with previous studies and international guidelines, a change of ≥0.5% in HbA1c was considered clinically meaningful, as this threshold has been shown to predict adverse outcomes and is widely used in diabetes trials [20,21,22]. The area under the curve (AUC), optimal cut-off values (based on Youden’s index), sensitivity, specificity, and 95% confidence intervals were reported for each predictor. ROC curves were plotted to visualize the discriminative capacity of the tested parameters. All statistical tests were two-tailed, and a significance threshold of *p* < 0.05 was used throughout.

## 3. Results

Demographic and clinical characteristics of the participants are shown in Table 1. The study included 550 patients with type 2 diabetes mellitus (T2DM), with a mean age of 56.2 ± 11.0 years; 43.3% were male (*n* = 238) and 56.7% female (*n* = 312). The majority were Turkish citizens (93.3%), while 6.7% were Syrian. Regarding residence, 45.8% lived in central Gaziantep and 54.2% in Islahiye, one of the most severely affected districts. The most common comorbidities were hyperlipidemia (56.5%), obesity (33.5%), and hypertension (30.9%). Diabetes duration exceeded 10 years in 45.6% of patients, was 5–10 years in 35.6%, and was less than 5 years in 18.7%. In terms of treatment, 58.4% used oral antidiabetic drugs, 17.5% insulin only, and 24.2% a combination of both (Table 1).

Comparison of mean biochemical parameters before and after the earthquake are shown in Table 2. Mean fasting plasma glucose levels increased from 167.4 ± 71.8 mg/dL to 178.2 ± 79.4 mg/dL (*p* < 0.001), and HbA1c levels rose significantly from 8.34 ± 1.9% to 8.87 ± 5.5% (*p* = 0.012). WBC count and PLT count also showed statistically significant elevations post-earthquake (*p* = 0.003 and *p* < 0.001, respectively). Conversely, levels of several parameters decreased significantly after the earthquake. Serum triglyceride levels dropped from 202.5 ± 170.4 mg/dL to 180.6 ± 113.1 mg/dL (*p* = 0.010), while HDL cholesterol levels declined from 53.2 ± 46.4 mg/dL to 45.1 ± 10.7 mg/dL (*p* < 0.001). Additionally, ALT levels were significantly lower post-earthquake (*p* = 0.002). No statistically significant differences were observed in creatinine, albumin, total cholesterol, LDL, hemoglobin, or inflammatory markers such as CRP and ESR (Table 2).

Associations of laboratory parameter changes with ΔHbA1c in other laboratory parameters are shown in Table 3. A moderate positive correlation was observed between ΔHbA1c and both fasting glucose (r = 0.362, *p* < 0.001) and triglyceride levels (r = 0.323, *p* < 0.001). ΔHbA1c showed weak but statistically significant positive correlations with total cholesterol (r = 0.107, *p* = 0.032) and LDL cholesterol (r = 0.173, *p* < 0.001). Conversely, negative correlations were found between ΔHbA1c and several protective or regulatory markers. A moderate inverse relationship was identified with serum albumin (r = −0.332, *p* = 0.029). Additionally, ΔHbA1c demonstrated weak but statistically significant negative correlations with HDL cholesterol (r = −0.175, *p* < 0.001) and white blood cell count (r = −0.112, *p* = 0.009). No significant correlations were observed between ΔHbA1c and hemoglobin, urea, creatinine, liver enzymes (ALT), or markers of inflammation such as CRP and ESR (Table 3). These correlation results represent the univariate analyses, and parameters with statistically significant associations were then entered into the multivariable regression model.

Multiple Linear Regression Analysis for Predictors of Change in HbA1c (ΔHbA1c) after the earthquake is shown in Table 4. In the initial biochemical model (Model 1), the regression was statistically significant (F(5, 544) = 17.16, *p* < 0.001) and explained approximately 29.8% of the variance in ΔHbA1c (Adjusted R^2^ = 0.284). In this model, increases in fasting glucose (β = 0.008, *p* < 0.001) and triglyceride levels (β = 0.005, *p* < 0.001) were significant positive predictors of increased HbA1c, whereas higher HDL (β = −0.011, *p* = 0.007), albumin (β = −0.017, *p* = 0.004), and WBC count (β = −0.010, *p* = 0.019) were independently associated with smaller increases in HbA1c. After adjustment for demographic and clinical covariates (Model 2: age, sex, BMI, diabetes duration, and insulin use), the regression remained statistically significant (F(11, 538) = 22.45, *p* < 0.001) and explained a greater proportion of variance in ΔHbA1c (Adjusted R^2^ = 0.319). In the fully adjusted model, ΔFasting Glucose (β = 0.007, *p* < 0.001) and ΔTriglyceride (β = 0.004, *p* = 0.001) continued to be significant independent predictors, while ΔHDL (β = −0.010, *p* = 0.011), ΔAlbumin (β = −0.016, *p* = 0.007), and ΔWBC (β = −0.009, *p* = 0.022) retained their protective associations. In addition, BMI (β = 0.006, *p* = 0.045), diabetes duration >10 years (β = 0.094, *p* = 0.009), and insulin use (β = 0.121, *p* = 0.003) emerged as significant predictors of higher ΔHbA1c. Age, sex, and diabetes duration of 5–10 years did not reach statistical significance (Table 4).

**Figure 2:**
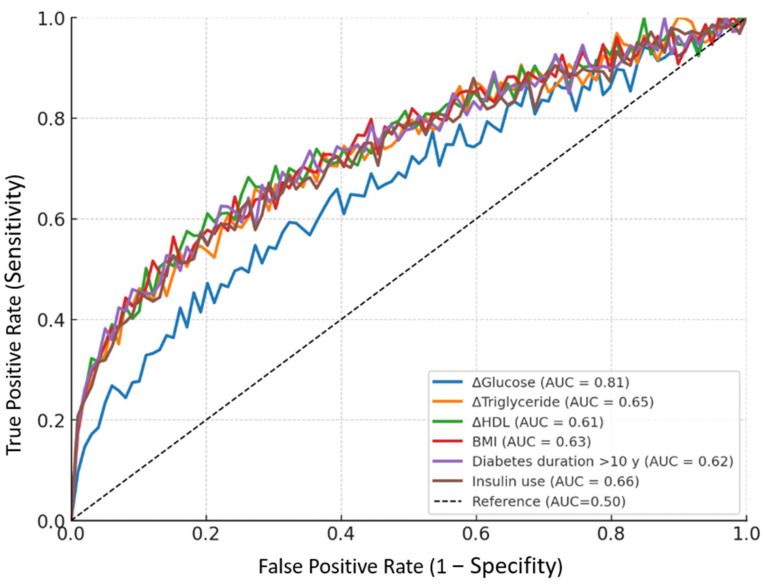
ROC curves for predicting glycemic worsening after earthquake.

ROC analysis was performed to evaluate the predictive performance of both biochemical and clinical parameters in identifying patients with post-earthquake glycemic worsening, defined as an increase in HbA1c greater than 0.5%. Among the tested variables, the change in fasting glucose (ΔGlucose) exhibited the highest discriminative ability and was identified as the strongest predictor of worsening glycemic control (AUC = 0.81, 95% CI: 0.74–0.84; *p* < 0.001). A cut-off value of >12.5 mg/dL yielded a sensitivity of 82.1% and specificity of 73.4%. The change in triglyceride levels (ΔTriglyceride) also demonstrated statistically significant predictive value, with an AUC of 0.65 (95% CI: 0.59–0.71; *p* < 0.001), and a cut-off of >25.0 mg/dL, offering 70.3% sensitivity and 61.8% specificity. The change in HDL levels (ΔHDL) showed modest predictive capacity, with an AUC of 0.61 (95% CI: 0.55–0.69; *p* = 0.016), and a cut-off of <−3.0 mg/dL, achieving 62.5% sensitivity and 55.2% specificity. Here, ΔHDL was defined as the difference between post- and pre-earthquake HDL levels; thus, negative values reflect decreases in HDL concentration. Beyond biochemical predictors, clinical parameters also contributed to risk stratification. BMI demonstrated an AUC of 0.63 (95% CI: 0.57–0.69; *p* = 0.002), with a cut-off of >29.0 kg/m^2^ providing 68.2% sensitivity and 60.7% specificity. Diabetes duration >10 years yielded an AUC of 0.62 (95% CI: 0.56–0.68; *p* = 0.004), while insulin use (any vs. none) showed an AUC of 0.66 (95% CI: 0.60–0.72; *p* < 0.001) (Table 5, Figure 2).

## 4. Discussion

This study evaluated the impact of a major natural disaster—the 6 February 2023 Kahramanmaraş-centered earthquakes—on glycemic control in adults with T2DM. Our findings demonstrated that both HbA1c and fasting glucose levels significantly increased following the earthquake, indicating deterioration in metabolic control. Moreover, we identified significant associations between changes in HbA1c and several laboratory parameters, particularly glucose, triglycerides, HDL, and albumin levels. Through linear regression and ROC analyses, ΔGlucose emerged as the strongest independent predictor of glycemic worsening. While our findings indicate significant deterioration in glycemic parameters after the earthquake, it is important to emphasize that poor diabetes management alone can also contribute to metabolic worsening. Therefore, the observed changes likely reflect a combined effect of pre-existing management challenges and disaster-related stressors, rather than stress alone.

These results were consistent with several studies conducted in disaster settings. Watanabe et al. investigated the metabolic consequences of the 2018 Northern Osaka Earthquake and reported a sustained increase in HbA1c levels over a three to six month period post-disaster [11]. Tanaka et al. demonstrated that individuals with impaired insulin secretion exhibited greater glycemic instability after the 2011 Great East Japan Earthquake [15]. Likewise, Kondo et al. [23] examined the impact of the 2016 Kumamoto Earthquake on glycemic control in patients with diabetes and reported significant increases in HbA1c, especially among insulin-treated and elderly individuals. Their findings highlighted the heightened vulnerability of older adults and those living alone, with stress, limited healthcare access, and disruption of daily routines being key contributing factors [23]. These mechanisms are consistent with our observations, in which worsening glycemic markers following the Kahramanmaraş earthquakes likely reflected the combined effects of physiological stress and healthcare system disruptions.

Fonseca et al. assessed the aftermath of Hurricane Katrina and reported that patients with diabetes experienced a marked rise in HbA1c, largely attributed to medication discontinuation, dietary disruptions, and psychological stress [13]. Likewise, Odhaib et al. documented similar trends during flood crises in Pakistan, emphasizing that carbohydrate-heavy food aid and insulin shortages contributed to metabolic decompensation [24]. Our findings mirrored these mechanisms, as the rise in glucose and triglyceride levels in our cohort may have reflected both dietary shifts and endocrine responses to acute stress. In addition, limited access to healthcare, irregular follow-up visits, and disrupted medication supplies likely contributed to glycemic deterioration. Supporting this, Nishikawa et al. [25] analyzed a case series of type 2 diabetes patients after the 2011 Great East Japan Earthquake and found that patients who continued regular hospital visits maintained better glycemic control than those with disrupted care. Their findings emphasized the importance of uninterrupted access to healthcare and continuity of care during disaster recovery phases [25]. These observations were consistent with our findings, where worsening glycemic markers may have partially resulted from barriers in accessing medication, clinical monitoring, and routine laboratory testing in the post-earthquake period.

From a pathophysiological perspective, Fujihara et al. proposed that stress-induced activation of the hypothalamic–pituitary–adrenal axis resulted in elevated cortisol and catecholamines, which promoted hepatic gluconeogenesis and insulin resistance [18]. This mechanism likely underpinned the observed increases in glycemia and supported our correlation between ΔHbA1c and ΔGlucose (r = 0.362, *p* < 0.001). The moderate inverse relationship with albumin (r = −0.332, *p* = 0.029) might also have suggested that inflammation, stress-related catabolism, or poor nutritional intake contributed to worsened glycemic profiles. Ciocca et al. [26] conducted a study after the 2009 L’Aquila earthquake in Italy, assessing psychological stress and its impact on patients with type 2 diabetes. They found that individuals with higher levels of post-traumatic stress disorder (PTSD) symptoms exhibited significantly poorer glycemic control and more maladaptive coping strategies. Their psychometric assessment underscored that psychological burden—not only physiological disruption—played a central role in post-disaster metabolic imbalance [26]. These findings were consistent with our results and reinforced the likelihood that psychological stress may have contributed to the worsening of glycemic markers in our cohort, even though we did not directly assess PTSD or coping behaviors.

In a broader context, Edelhoff et al. investigated adults who had experienced earthquake stress in early life and found that this exposure significantly increased the risk of developing a metabolic syndrome later in life, particularly among those engaged in night shift work [27]. This study highlighted the long-term metabolic consequences of early trauma and its interaction with adverse lifestyle factors, further supporting the hypothesis that disaster-induced stress has both immediate and lasting implications for glucose and lipid metabolism. Our findings, although focused on short-term glycemic outcomes, align with this notion of stress-related metabolic vulnerability.

Our multiple linear regression analysis identified ΔGlucose, ΔTriglyceride, ΔHDL, and ΔAlbumin as independent predictors of post-disaster glycemic deterioration. These results were in line with findings from Tanaka et al. and Hirai et al., who reported that individuals with higher triglyceride levels and lower HDL concentrations were more likely to experience metabolic disruption after earthquakes [14,28]. It should be noted that these changes were observed within the three to five-month post-disaster period, which corresponds to the timeframe necessary for HbA1c to capture medium-term glycemic deterioration. Furthermore, ROC analysis in our study revealed that ΔGlucose had the highest discriminative power (AUC = 0.81), suggesting its clinical utility in the early identification of patients at risk for post-disaster hyperglycemia.

Our study demonstrated that higher WBC counts were inversely associated with post-disaster HbA1c increases. This finding may reflect the role of leukocytes as markers of systemic inflammation and stress response. Elevated WBC has been linked to insulin resistance and adverse metabolic outcomes in previous epidemiological studies [29,30]. However, the negative association observed in our cohort may indicate that patients with higher WBC counts were more likely to have received closer clinical monitoring or presented acute-phase responses that did not translate into sustained hyperglycemia. Similar paradoxical findings have been reported in disaster-related cohorts, where acute inflammatory responses did not always correlate with long-term glycemic worsening [31].

In addition, serum albumin showed a significant inverse correlation with HbA1c changes. Hypoalbuminemia is frequently associated with malnutrition, systemic inflammation, and oxidative stress, all of which can impair metabolic stability [32]. Lower albumin concentrations have previously been reported as predictors of poor glycemic control and adverse outcomes in diabetic patients [33]. Our results are consistent with these observations, suggesting that reduced albumin may serve as a surrogate marker of both nutritional compromise and stress-induced catabolism in disaster settings. These findings highlight the potential utility of routinely measured laboratory markers such as albumin in identifying patients at greater risk of metabolic deterioration when healthcare resources are disrupted.

While previous studies have predominantly focused on pediatric populations or individuals with type 1 diabetes [12,19], our study contributes novel insights by specifically examining adult patients with type 2 diabetes mellitus (T2DM)—the most prevalent form of diabetes worldwide. Notably, Tarçın et al. reported that continuous glucose monitoring (CGM) devices played a protective role in maintaining glycemic stability among children with type 1 diabetes following the Kahramanmaraş earthquakes [12]. In contrast, our study demonstrated that even in the absence of advanced monitoring technologies such as CGM, routine laboratory parameters—particularly HbA1c and fasting glucose—were sufficient to detect significant metabolic deterioration in adult T2DM patients. This distinction emphasizes the broader applicability of our findings to resource-limited settings where CGM is not routinely available, highlighting the utility of basic biochemical follow-up in post-disaster care for adults with chronic conditions.

Unlike previous reports that predominantly focused on type 1 diabetes or pediatric populations, our study uniquely investigates adults with T2DM—the most prevalent diabetic group worldwide—in the context of the devastating Kahramanmaraş earthquakes. Moreover, by integrating routine biochemical markers with advanced statistical analyses such as multiple regression and ROC curves, this research provides novel insights into metabolic vulnerability and offers practical tools for disaster-related risk stratification.

These findings have important implications for disaster preparedness. In post-disaster settings, where resources are constrained, simple and widely available markers such as fasting glucose can be rapidly measured and used in triage to identify patients at risk of metabolic deterioration. In addition, incorporating clinical characteristics such as insulin therapy, long diabetes duration, and obesity into disaster response protocols could enable targeted monitoring and prioritization of high-risk individuals. Integrating these strategies into national disaster preparedness plans may help mitigate adverse outcomes for patients with chronic conditions such as diabetes.

### Limitations of the Study

This study has some limitations. First, its retrospective pre–post design limited the ability to establish causal relationships between the earthquake and changes in glycemic parameters, and pre-existing trends were not modeled. Second, data were obtained from two centers in a single region, which may limit generalizability to populations with different healthcare systems or socioeconomic contexts. Third, behavioral and psychosocial factors such as stress, dietary intake, and medication access were not directly assessed, and potential confounders including adherence, treatment continuity, and mental health impacts could not be fully accounted for. Fourth, although CBC data were available, we did not analyze derived indices such as the neutrophil-to-lymphocyte ratio (NLR), which might have provided additional insights into stress-related inflammation; this represents a limitation that future studies should address. Fifth, detailed information on diabetes-related complications (e.g., nephropathy, neuropathy) and comorbidities was not consistently available and thus could not be included in multivariable analysis. Although both laboratories applied standardized procedures, minor inter-instrument variability cannot be excluded, though our paired design reduces this risk. Finally, our definition of glycemic worsening (ΔHbA1c >0.5%), although consistent with prior trials (UKPDS, ADVANCE) and ADA recommendations, may not capture all clinically relevant changes. Despite these limitations, the large sample size and detailed biochemical profiling enhance the robustness and clinical relevance of the findings.

Both centers included in this study operate under the Gaziantep Provincial Health Directorate, adhere to the same national health insurance system, and follow standardized protocols for laboratory testing and clinical care. Therefore, patient populations are broadly comparable in terms of socio-economic status and healthcare standards. Nonetheless, minor variations in referral patterns or case-mix between a tertiary training hospital and a district hospital cannot be completely excluded, which represents a potential limitation.

## 5. Conclusions

In conclusion, this study demonstrated that glycemic control became significantly worse in patients with type 2 diabetes mellitus following the Kahramanmaraş-centered earthquakes of 6 February 2023. Both HbA1c and fasting glucose levels increased notably in the post-disaster period, underscoring the vulnerability of individuals with chronic metabolic conditions during natural catastrophes. Biochemical markers such as ΔGlucose, ΔTriglyceride, ΔHDL, and ΔAlbumin were significantly associated with changes in glycemic status. Among these, ΔGlucose emerged as the most powerful predictor, as confirmed by both regression and ROC analyses. This study adds originality by focusing on adult T2DM patients—rather than the more frequently studied type 1 or pediatric populations—and by integrating comprehensive biochemical profiling with advanced statistical approaches. These aspects provide novel insights into disaster-related metabolic vulnerability and highlight the utility of simple, routine laboratory parameters as predictive tools. These findings suggest that routine laboratory parameters may serve as practical and accessible tools for identifying patients at risk of metabolic deterioration during crises. Given the well-established biological relationship between HbA1c and fasting glucose, some degree of correlation was expected. However, by analyzing changes (Δ values) rather than absolute measures, and confirming the associations in multivariable models, we aimed to minimize redundancy, although this overlap remains an inherent limitation. Given the increasing frequency of climate-related disasters and humanitarian emergencies globally, integrating chronic disease management—particularly for diabetes—into disaster preparedness and response plans is essential. Future prospective, multicenter studies with behavioral and psychosocial assessments are warranted to develop comprehensive risk stratification models and targeted interventions for vulnerable populations.

## Figures and Tables

**Table 1:** Demographic and clinical characteristics of the participants (*N* = 550).

Variable	Mean ± SD, *n* (%)
Age (years)	56.2 ± 11.0
Gender	
Male	238 (43.3%)
Female	312 (56.7%)
Nationality	
Turkish	513 (93.3%)
Syrian	37 (6.7%)
Place of Residence	
Central Gaziantep	252 (45.8%)
Islahiye	298 (54.2%)
Comorbidities	
Hypertension	170 (30.9%)
Hyperlipidemia	311 (56.5%)
Coronary artery disease	26 (4.7%)
Obesity (BMI > 30 kg/m^2^)	184 (33.5%)
Chronic kidney disease	39 (7.1%)
Duration of T2DM (years)	
<5 years	103 (18.7%)
5–10 years	196 (35.6%)
>10 years	251 (45.6%)
Treatment Modality	
Oral antidiabetics	321 (58.4%)
Insulin therapy	96 (17.5%)
Combined (oral + insulin)	133 (24.2%)

**Table 2:** Comparison of mean biochemical parameters before and after the earthquake (*N* = 550).

**Parameter**	**Pre-Earthquake** **(Mean ± SD)**	**Post-Earthquake** **(Mean ± SD)**	***p*** **-Value**
White Blood Cell (WBC) (×10^3^/μL)	8.49 ± 5.1	9.42 ± 5.6	0.003 **
Hemoglobin (g/dL)	14.2 ± 1.8	13.9 ± 2.1	0.321
Platelet (PLT) (×10^3^/μL)	272.3 ± 88.7	292.8 ± 87.1	<0.001 **
Urea (mg/dL)	32.8 ± 5.8	30.9 ± 13.5	0.023 *
Creatinine (mg/dL)	1.25 ± 7.1	0.81 ± 0.3	0.181
ALT (U/L)	23.6 ± 16.5	20.9 ± 14.6	0.002 **
Albumin (g/dL)	27.5 ± 17.5	28.8 ± 18.4	0.150
Total Cholesterol (mg/dL)	190.6 ± 45.7	193.5 ± 43.7	0.239
LDL (mg/dL)	106.2 ± 46.3	108.7 ± 44.9	0.354
Triglyceride (TG) (mg/dL)	202.5 ± 170.4	180.6 ± 113.1	0.010 *
HDL (mg/dL)	53.2 ± 46.4	45.1 ± 10.7	<0.001 **
Glucose (mg/dL)	167.4 ± 71.8	178.2 ± 79.4	<0.001 **
HbA1c (%)	8.34 ± 1.9	8.87 ± 5.5	0.012 *
ESR (mm/h)	22.8 ± 36.7	16.9 ± 21.1	0.636
CRP (mg/L)	7.58 ± 10.8	7.18 ± 17.5	0.863
Vitamin D (ng/mL)	14.3 ± 18.1	15.1 ± 18.0	0.191
Vitamin B12 (pg/mL)	421.9 ± 369.9	395.4 ± 228.6	0.499
TSH (μIU/mL)	2.99 ± 4.6	2.36 ± 2.0	0.192

Differences between pre- and post-earthquake values were analyzed using paired samples *t*-test (confirmed by Wilcoxon signed-rank test). * Statistically significant at *p* < 0.05; ** highly significant at *p* < 0.001.

**Table 3:** Associations of changes in laboratory parameters with ΔHbA1c (*N* = 550).

Parameter	Correlation Coefficient (r)	*p*-Value
White Blood Cell (WBC)	−0.112	0.009 **
Hemoglobin	0.053	0.240
Platelet (PLT)	−0.064	0.157
Urea	0.035	0.451
Creatinine	0.040	0.384
ALT	0.002	0.973
Albumin	−0.332	0.029 *
Total Cholesterol	0.107	0.032 *
LDL	0.173	<0.001 **
Triglyceride (TG)	0.323	<0.001 **
HDL	−0.175	<0.001 **
Glucose	0.362	<0.001 **
ESR	0.116	0.706
CRP	−0.057	0.643
Vitamin D	−0.535	0.137
Vitamin B12	−0.046	0.689
TSH	0.050	0.659

Spearman’s rho correlation test was used. * Statistically significant at *p* < 0.05; ** highly significant at *p* < 0.001.

**Table 4:** Multiple linear regression analysis for predictors of change in HbA1c (ΔHbA1c) after the earthquake.

Independent Variable	Unstandardized β	Standard Error (SE)	Standardized β (Beta)	t-Value	*p*-Value
Age (years)	0.002	0.001	0.065	1.92	0.055
Sex (male vs. female)	−0.048	0.032	−0.058	−1.50	0.135
BMI (kg/m^2^)	0.006	0.003	0.082	2.01	0.045 *
Diabetes duration 5–10 y (vs. <5 y)	0.051	0.028	0.067	1.83	0.068
Diabetes duration >10 y (vs. <5 y)	0.094	0.036	0.101	2.61	0.009 **
Insulin use (any vs. none)	0.121	0.041	0.112	2.95	0.003 **
ΔFasting Glucose (mg/dL)	0.008	0.002	0.256	4.12	<0.001 **
ΔTriglyceride (mg/dL)	0.005	0.001	0.221	3.84	<0.001 **
ΔHDL (mg/dL)	−0.011	0.004	−0.142	−2.71	0.007 *
ΔAlbumin (g/dL)	−0.017	0.006	−0.168	−2.94	0.004 *
ΔWBC (×10^3^/μL)	−0.010	0.003	−0.124	−2.36	0.019 *
Constant (Intercept)	0.342	0.092	—	3.72	<0.001 **

Multiple linear regression analysis was performed using ΔHbA1c as the dependent variable. Model Summary: R^2^ = 0.298, Adjusted R^2^ = 0.284, F(5, 544) = 17.16, *p* < 0.001. * Statistically significant at *p* < 0.05; ** highly significant at *p* < 0.001.

**Table 5:** ROC analysis for predicting glycemic worsening.

Parameter	Cut-Off	Sensitivity	Specifity	AUC (95% CI)	*p* Value
ΔGlucose (mg/dL)	>12.5	82.1%	73.4%	0.81 (0.74–0.84)	<0.001 **
ΔTriglyceride (mg/dL)	>25.0	70.3%	61.8%	0.65 (0.59–0.71)	<0.001 **
ΔHDL (mg/dL)	<−3.0	62.5%	55.2%	0.61 (0.55–0.69)	0.016 *
BMI (kg/m^2^)	>29.0	68.2%	60.7%	0.63 (0.57–0.69)	0.002 **
Diabetes duration >10 y (vs. <5 y)	—	65.4%	59.1%	0.62 (0.56–0.68)	0.004 **
Insulin use (any vs. none)	—	71.7%	64.3%	0.66 (0.60–0.72)	<0.001 **

* Statistically significant at *p* < 0.05; ** highly significant at *p* < 0.001.

## Data Availability

The raw data supporting the conclusions of this article will be made available by the corresponding author on request.
